# Increased expression of SUMO1P3 predicts poor prognosis and promotes tumor growth and metastasis in bladder cancer

**DOI:** 10.18632/oncotarget.6946

**Published:** 2016-01-19

**Authors:** Yonghao Zhan, Yuchen Liu, Chaoliang Wang, Junhao Lin, Mingwei Chen, Xiaoying Chen, Chengle Zhuang, Li Liu, Wen Xu, Qing Zhou, Xiaojuan Sun, Qiaoxia Zhang, Guoping Zhao, Weiren Huang

**Affiliations:** ^1^ Key Laboratory of Medical Reprogramming Technology, Shenzhen Second People's Hospital, The First Affiliated Hospital of Shenzhen University, Shenzhen, China; ^2^ Department of Urology, Peking University First Hospital, Institute of Urology, Peking University, National Urological Cancer Centre, Beijing, China; ^3^ Shantou University Medical College, Shantou, China; ^4^ Urology Department, The First Affiliated Hospital of Zhengzhou University, Zhengzhou, China; ^5^ Shanghai-MOST Key Laboratory of Health and Disease Genomics, Chinese National Human Genome Centerat Shanghai, Shanghai, China

**Keywords:** SUMO1P3, bladder cancer, tumor growth, tumor metastasis

## Abstract

Bladder cancer is one of the most common malignancies worldwide. Long non-coding RNAs (lncRNAs) are a class of non-coding RNAs that play crucial roles in diverse biological processes. The pseudogene-expressed lncRNA is one major type of lncRNA family. Small ubiquitin-like modifier (SUMO) 1 pseudogene 3, (SUMO1P3) is a novel indentified lncRNA that was previously reported to be up-regulated in gastric cancer. However, we know nothing about the biological function and underlying mechanism of SUMO1P3 in tumor. Furthermore, the relationship between SUMO1P3 and bladder cancer is completely unknown. We hypothesized that SUMO1P3 also have roles in bladder cancer.

In this study, we found that SUMO1P3 was significantly up-regulated in bladder cancer tissues compared with paired-adjacent nontumorous tissues in a cohort of 55 bladder cancer patients. Moreover, up-regulated SUMO1P3 expression was positively correlated with greater histological grade (P<0.05) and advanced TNM stage (P<0.05). Furthermore, we found cell proliferation / migration inhibition and apoptosis induction were also observed in SUMO1P3 siRNA-transfected bladder cancer cells. Our data suggest that SUMO1P3 plays oncogenic roles in bladder cancer and can be used as a potential prognostic and therapeutic target.

## INTRODUCTION

Bladder cancer is the most common genitourinary tumors worldwide, and its incidence and mortality have been significantly increased in the past decades [1, 2]. However, at the early stage of bladder cancer there are no specific symptoms for these patients [3]. Since the prognosis of bladder cancer is closely related to the stage of disease at diagnosis, novel diagnostic markers for early stage are urgently needed [4, 5]. Despite improvements in surgery and adjuvant chemoradiotherapies, the 5-year survival rate for patients with bladder cancer remains at only 50–C60 % [6, 7].

The rapid development of RNA genomics has highlighted the role of long non-coding RNAs (lncRNAs) in many human diseases, especially in cancers [8–10]. Recent evidence showed that lncRNAs play important regulatory roles in diverse biological processes, such as transcriptional regulation, cell growth and tumorigenesis [11–14]. Examples include HOTAIR in breast cancer, MALAT1 in lung cancer and PCAT-1 in prostate cancer, indicating that lncRNAs play crucial roles in tumorigenesis or tumor progression [15–17]. Small ubiquitin-like modifier 1 pseudogene 3 (SUMO1P3) is a novel indentified long non-coding RNA that was originally identified as a potential prognostic and therapeutic target for gastric cancer [18]. However, the relationship between lncRNA SUMO1P3 and bladder cancer is completely unknown.

In the present study, we found that lncRNA SUMO1P3 was significantly up-regulated in bladder cancer tissues compared with paired-adjacent nontumorous tissues in a cohort of 55 bladder cancer patients. Further experiments indicated that silencing lncRNA SUMO1P3 could inhibit proliferation, induce apoptosis and suppress migration of the bladder cancer cell lines.

## RESULTS

### SUMO1P3 was up-regulated in bladder cancer

**Figure 1:**
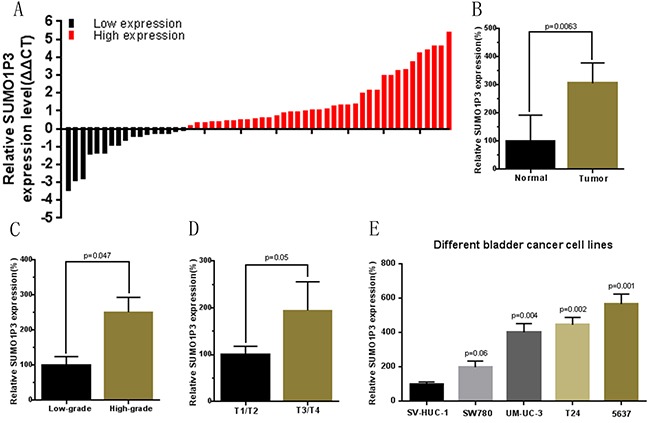
The long noncoding RNA SUMO1P3 was up-regulated in bladder cancer The relative expression levels of SUMO1P3 were detected using Real-Time qPCR. **A.** SUMO1P3 expression levels were higher in bladder cancer tissues than those in normal bladder tissues. **B.** The relative expression level of SUMO1P3 was significantly higher in bladder cancer tissues compared with matched normal tissues. **C.** SUMO1P3 expression levels was significantly higher in patients with a higher pathological stage. **D.** SUMO1P3 expression levels were significantly higher in patients with higher TNM stage. **E.** SUMO1P3 expression levels were higher in bladder cancer cell lines than those in normal urothelial cell line. Data are shown as mean ± SD.

The relative expression level of SUMO1P3 was determined by using Real-Time qPCR in a total of 55 patients with urothelial bladder cancer and different cell lines. The SUMO1P3 expression fold change (bladder cancer tissue / matched normal tissue) in each patient was indicated in Figure 1A. As shown in Figure 1B, SUMO1P3 was up-regulated in bladder cancer tissues compared to pair-matched adjacent normal tissues. Moreover, up-regulated SUMO1P3 expression was positively correlated with greater histological grade (Figure 1C) and advanced TNM stage (Figure 1D). Furthermore, SUMO1P3 was up-regulated in bladder cancer cell lines (Figure 1E) compared to normal urothelial cell line. These results indicated that SUMO1P3 should play oncogenic roles in bladder cancer. Clinicopathological features of 55 patients and statistical results are shown in Table 1 and Table 2, respectively.

**Table 1:** Correlation between SUMO1P3 expression and clinicopathological features of UCB patients

Parameters Total	Group	Total	SUMO1P3 expression		*P* value
			High	Low	
**Gender**	Male	40(73%)	28(51%)	12(22%)	0.528
	Female	15(27%)	10(18%)	5(9%)	
**Age (years)**	< 60	20(36%)	14(25%)	6(11%)	0.580
	≥ 60	35(64%)	24(44%)	11(20%)	
**Tumor size (cm)**	< 3 cm	21(38%)	12(22%)	9(16%)	0.114
	≥ 3 cm	34(62%)	26(47%)	8(15%)	
**Multiplicity**	Single	33(60%)	26(47%)	7(13%)	0.054
	Multiple	22(40%)	12(22%)	10(18%)	
**Histological grade**	Low(G1)	23(42%)	11(20%)	12(22%)	0.005
	High(G2,G3)	32(58%)	27(49%)	5(9%)	
**Tumor stage (T)**	T1,T2	38(69%)	23(42%)	15(27%)	0.037
	T3,T4	17(31%)	15(27%)	2(4%)	
**Lymph nodes metastasis (N)**	NO	53(96%)	27(49%)	16(27%)	0.618
	YES	2(4%)	1(2%)	1(2%)	
**Distal metastasis (M)**	M0	55(100%)	38(69%)	17(31%)	
	M1	0	0	0	

**Table 2:** Summary of clinicopathological features of tissues of bladder cancer

Pt No.	Sex	Age	Stage	Grade	Pt No.	Sex	Age	Stage	Grade
**1**	M	66	T2bN0M0	H	29	M	63	T2aN0M0	L
**2**	F	38	T3aN0M0	H	30	M	58	T4aN0M0	H
**3**	F	64	T1N0M0	L	31	M	50	T2bN0M0	H
**4**	M	75	T2bN0M0	H	32	M	59	T4N0M0	H
**5**	M	58	T3aN0M0	H	33	F	62	T4aN0M0	H
**6**	M	65	T2bN0M0	H	34	M	41	T1N0M0	L
**7**	M	53	T1N0M0	L	35	M	62	T4aN0M0	H
**8**	M	59	T2bN0M0	H	36	M	76	T2bN0M0	L
**9**	M	43	T3aN0M0	H	37	M	25	T1N0M0	L
**10**	F	64	T2bN0M0	H	38	F	74	T3aN0M0	H
**11**	M	63	T2bN0M0	H	39	F	70	T1N0M0	L
**12**	M	72	T3aN0M0	H	40	F	72	T1N0M0	L
**13**	M	69	T1N0M0	L	41	M	73	T3bN0M0	H
**14**	M	68	T2bN0M0	H	42	M	63	T3aN0M0	H
**15**	F	63	T3aN0M0	H	43	M	46	T1N0M0	L
**16**	F	89	T1N0M0	L	44	M	57	T4aN0M0	H
**17**	M	78	T2aN0M0	L	45	M	70	T2bN0M0	H
**18**	M	70	T2aN0M0	L	46	M	77	T3aN0M0	H
**19**	F	41	T2aN0M0	L	47	M	66	T1N0M0	L
**20**	M	59	T2bN0M0	H	48	M	53	T2aN0M0	L
**21**	F	73	T2aN0M0	L	49	M	49	T1N0M0	L
**22**	M	67	T2bN0M0	H	50	M	47	T2aN0M0	L
**23**	F	61	T3aN0M0	H	51	M	68	T1N0M0	L
**24**	M	58	T4aN3M0	H	52	M	61	T3aN0M0	H
**25**	M	63	T2aN0M0	L	53	F	74	T2bN0M0	H
**26**	F	51	T1N0M0	L	54	F	60	T2aN0M0	H
**27**	M	86	T1N0M0	L	55	M	73	T2bN1M0	H
**28**	M	54	T2bN0M0	H					

### Specific siRNA down-regulated expression of SUMO1P3

**Figure 2:**
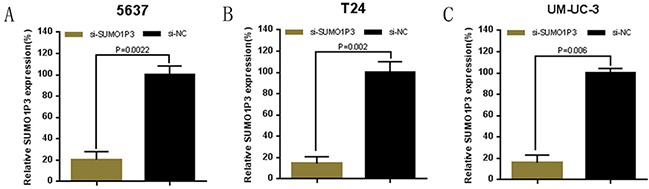
Expression changes of SUMO1P3 after transfection of SUMO1P3 specific siRNA or negative control siRNA The relative expression level was determined using real-time qPCR. The SUMO1P3 specific siRNA significantly down-regulated the expression level of SUMO1P3 in 5637 **A.** T24 **B.** and UM-UC-3 **C.** cells. Data are shown as mean ± SD.

Bladder cancer 5637, T24 and UM-UC-3 cells were cultured and transfected with SUMO1P3 siRNA or negative control siRNA. At 48 hours after transfection, the related expression level of SUMO1P3 was analyzed by qRT-PCR and the results of showed that the relative level of SUMO1P3 in 5637 (Figure 2A), T24 (Figure 2B) and UM-UC-3 (Figure 2C) cells was significantly down-regulated by the SUMO1P3 siRNA.

### Silencing SUMO1P3 inhibited cell proliferation

**Figure 3:**
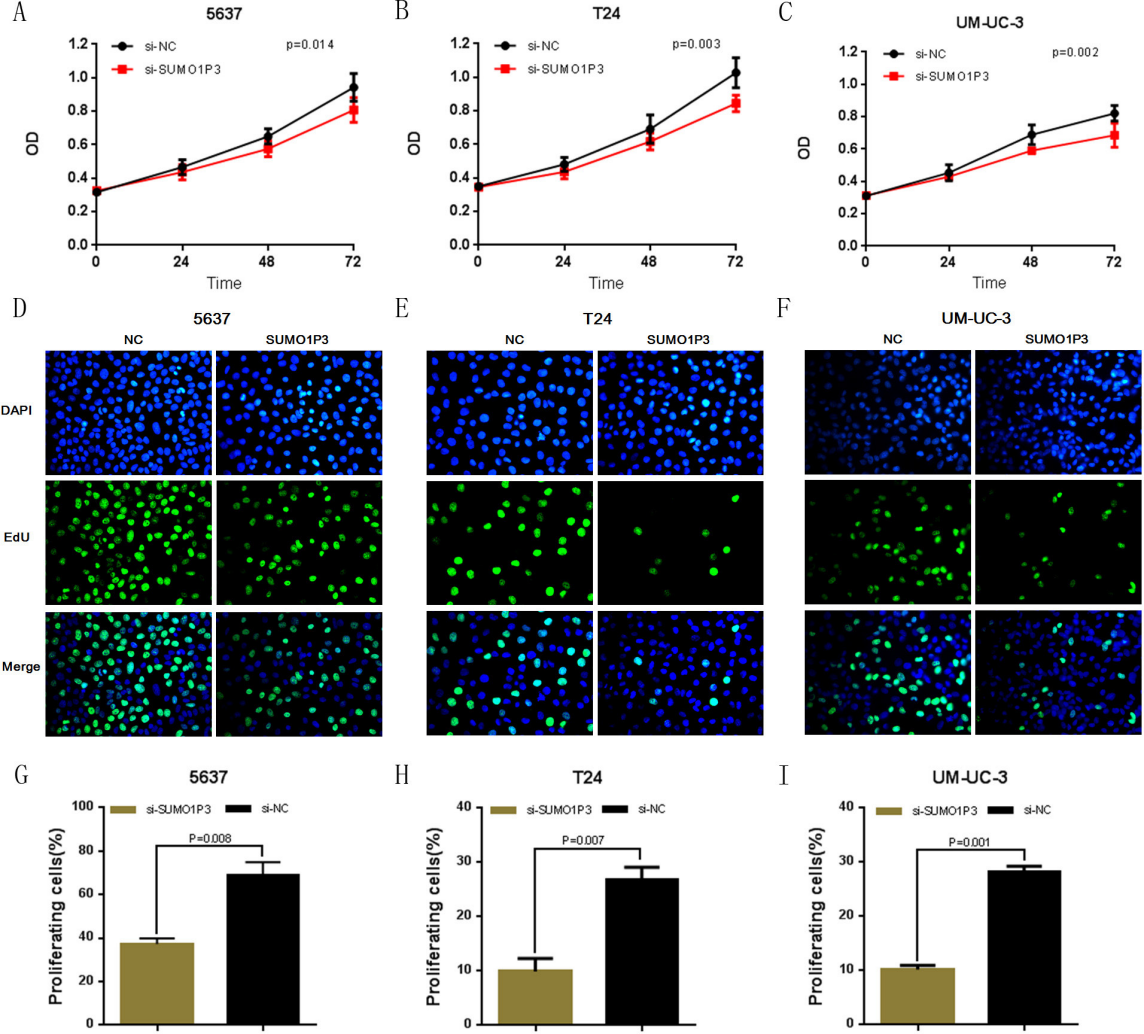
Silencing long noncoding RNA SUMO1P3 inhibited cell proliferation in bladder cancer cells Cell proliferation was determined by both CCK-8 assay and Edu assay. Cell proliferation inhibition was observed in bladder cancer 5637 **A, D.** and **G.** T24 **B, E.** and **H.** and UM-UC-3 **C, F.** and **I.** cells. Data are shown as mean ± SD.

We further determined whether SUMO1P3 promotes cell proliferation in bladder cancer. Bladder cancer 5637, T24 and UM-UC-3 cells were transfected with SUMO1P3 siRNA or negative control siRNA and the cell proliferation changes of bladder cells were determined using both CCK-8 assay and Edu assay. Cell growth arrest was observed in 5637 (Figure 3A, 3D and 3G), T24 (Figure 3B, 3E and 3H) and UM-UC-3 (Figure 3C, 3F and 3I) cells as expected. These results confirmed that SUMO1P3 increases cell proliferation in bladder cancer.

### Silencing SUMO1P3 induced apoptosis

**Figure 4:**
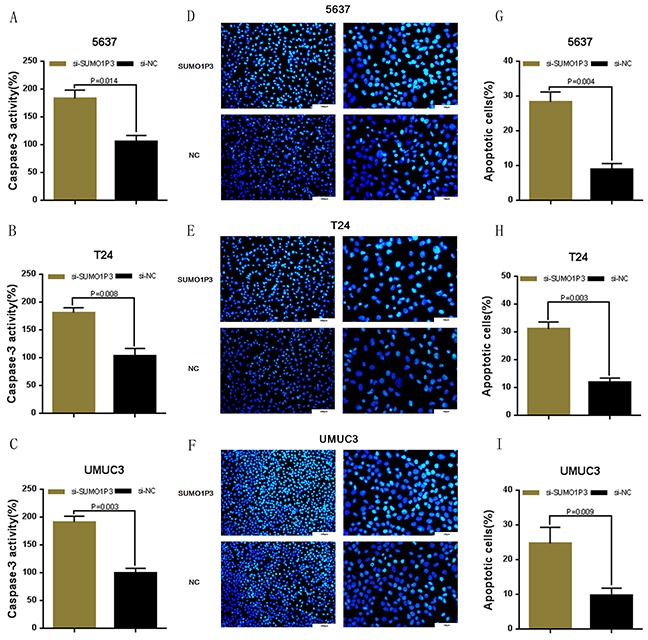
Silencing long noncoding RNA SUMO1P3 induced cell apoptosis in bladder cancer cells Cell apoptosis was determined by both ELISA assay and Hoechst 33342 staining assay. Induced cell apoptosis was observed in bladder cancer 5637 **A, D.** and **G.** T24 **B, E.** and **H.** and UM-UC-3 **C, F.** and **I.** cells. Data are shown as mean ± SD.

**Figure 5:**
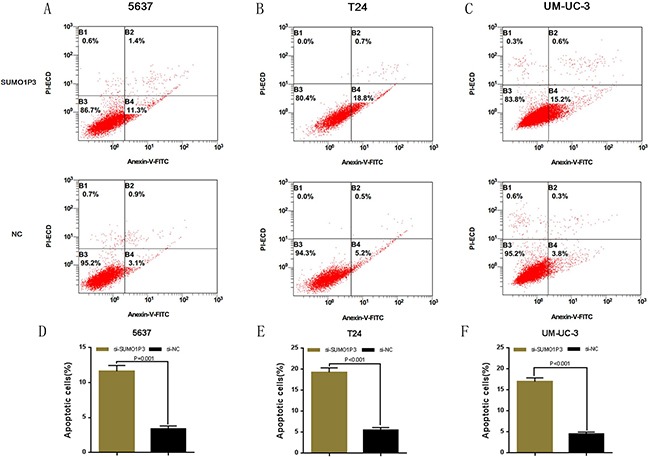
Silencing long noncoding RNA SUMO1P3 induced cell apoptosis in bladder cancer cells Cell apoptosis was also determined by Flow cytometry. Induced cell apoptosis was observed in bladder cancer 5637 **A.** and **D.** T24 **B.** and **E.** and UM-UC-3 **C.** and **F.** cells. Data are shown as mean ± SD.

We determined whether SUMO1P3 can inhibit cell apoptosis in bladder cancer. Bladder cancer 5637, T24 and UM-UC-3 cells were transfected with SUMO1P3 siRNA or negative control siRNA. The relative activity of caspase-3 was determined using ELISA assay (Figure 4A, 4B and 4C). Furthermore, the apoptosis ratio in bladder cancer cells was measured using Hoechst 33342 staining and Flow cytometry. Induced cell apoptosis was also observed in 5637 (Figure 4D, 4G and Figure 5A, 5D), T24 (Figure 4E, 4H and Figure 5B, 5E) and UM-UC-3 (Figure 4F, 4I and Figure 5C, 5F) cells. These results demonstrated that SUMO1P3 suppresses cell apoptosis in bladder cancer.

### Silencing SUMO1P3 inhibited cell migration

**Figure 6:**
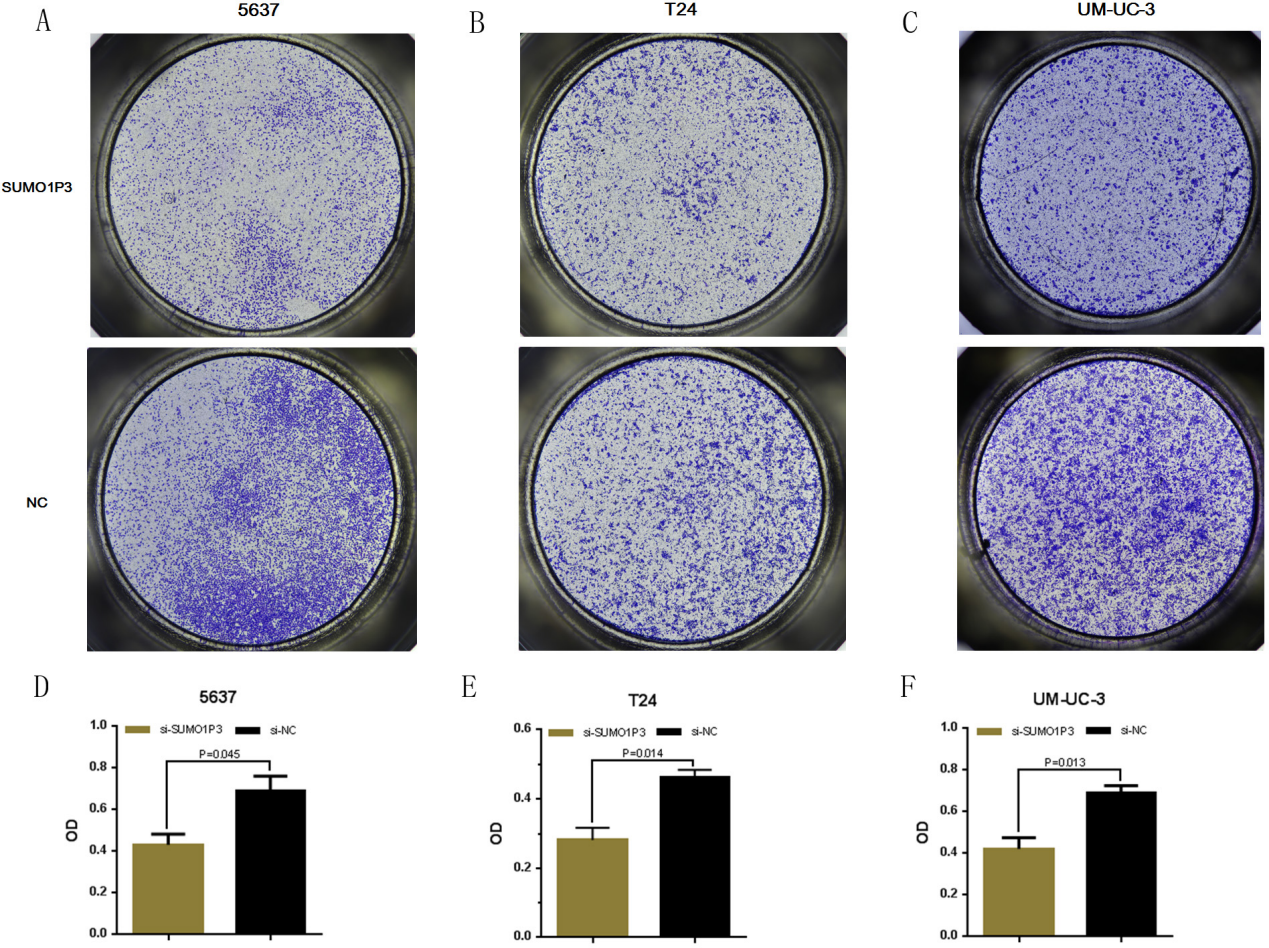
Silencing long noncoding RNA SUMO1P3 inhibited cell migration in bladder cancer cells Cell migration was determined by both wound healing assay. Cell migration inhibition was observed in bladder cancer 5637 **A.** and **D.** T24 **B.** and **E.** and UM-UC-3 **C.** and **F.** cells. Data are shown as mean ± SD.

**Figure 7:**
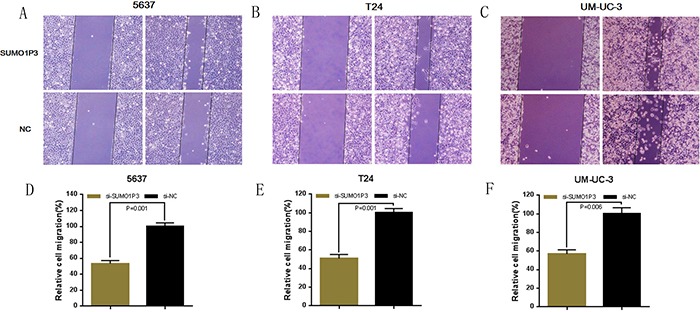
Silencing long noncoding RNA SUMO1P3 inhibited cell migration in bladder cancer cells Cell migration was also determined by transwell assay. Cell migration inhibition was observed in bladder cancer 5637 **A.** and **D.** T24 **B.** and **E.** and UM-UC-3 **C.** and **F.** cells. Data are shown as mean ± SD.

Finally, we determined whether SUMO1P3 promotes cell migration in bladder cancer. Bladder cancer 5637, T24 and UM-UC-3 cells were transfected with SUMO1P3 siRNA or negative control siRNA and the cell migration changes of bladder cells were determined by both wound healing assay and transwell assay. Cell migration arrest was observed in 5637 (Figure 6A, 6D and Figure 7A, 7D), T24 (Figure 6B, 6E and Figure 7B, 7E) and UM-UC-3 cells (Figure 6C, 6F and Figure 7C, 7F) as expected. These results confirmed that SUMO1P3 increases cell migration in bladder cancer.

## DISCUSSION

Bladder cancer is the most common genitourinary malignancies in human populations. The prognosis of bladder cancer remains quite poor, because most bladder cancers are found at advanced-stage when treatments are less effective [19, 20]. Thus, finding new molecular targets for bladder cancer diagnosis, prognosis and treatment has the potential to improve the clinical strategies and outcomes of bladder cancer [21, 22].

Long non-coding RNAs (lncRNAs) are a type of RNA molecule longer than 200 nucleotides and not translated into a protein [23, 24]. Recently, numerous pieces of evidence indicate that lncRNAs play a vital role in cancer progression and development [25, 26]. LncRNA SUMO1P3 was previously reported to be up-regulated in gastric cancer. However, the relationship between lncRNA SUMO1P3 and bladder cancer is completely unknown. Furthermore, we know nothing about the biological function and underlying mechanism of lncRNA SUMO1P3 in cancers [27].

To the best of our knowledge, this is the first report of lncRNA SUMO1P3 being involved in the development of bladder cancer. In this study, we found that lncRNA SUMO1P3 was up-regulated in bladder cancer compared with paired-adjacent nontumorous tissue and up-regulated SUMO1P3 expression was positively correlated with greater histological grade and advanced TNM stage. These results suggest that lncRNA SUMO1P3 may emerge as a novel player in the state of bladder cancer. In order to understand the biological functions of lncRNA SUMO1P3, we detected the cell proliferation, apoptosis and migration by silencing SUMO1P3 in the related bladder cancer cell lines. Inhibited proliferation, increased apoptosis and suppressed migration were observed in SUMO1P3 siRNA-transfected bladder cells. These findings indicated that SUMO1P3 may play key roles in the progression and development in bladder cancer.

In conclusion, the expression level of the lncRNA SUMO1P3 is increased in bladder cancer tissues compared with paired-adjacent nontumorous tissues. Up-regulated SUMO1P3 expression has been associated with poor prognosis, likely due to the ability of SUMO1P3 to induce cell growth and metastasis in bladder cancer cells. The molecular mechanism underlying SUMO1P3 upregulation in bladder cancer is still to be studied in future works.

Cumulatively, these findings indicate that SUMO1P3 play an oncogenic role in bladder cancer and SUMO1P3 may be used as a potential prognostic and therapeutic target of bladder cancer.

## MATERIALS AND METHODS

### Patients and clinical samples collection

A total of 55 urothelial bladder cancer tissues and their pair-matched adjacent normal tissues were obtained with informed consent from patients who underwent radical resections at Shenzhen Second People's Hospital, Shenzhen, China. This study was performed with the approval of the Research Ethics Committee of Shenzhen Second People's Hospital.

### Cell lines and cell culture

Bladder cancer cells lines (5637, T24, UM-UC-3, SW780) and SV-40-immortalized human uroepithelial cell line (SV-HUC-1) used in this study were purchased from the Institute of Cell Research, Chinese Academy of Sciences, Shanghai, China. The T24, UM-UC-3 and SV-HUC-1 cells were cultured in Dulbecco's Modified Eagle Medium (Invitrogen, Carlsbad, CA, USA) plus 10 % fetal bovine serum. The 5637 and SW780 cells were cultured in RPMI-1640 Medium (Invitrogen, Carlsbad, CA, USA) plus 10 % fetal bovine serum. Plates were then placed at 37°C with a humidified atmosphere of 5 % CO_2_ in incubator.

### siRNA transfection

Small interfering RNA that targeted SUMO1P3 and a scrambled negative control were purchased from GenePharma, Shanghai, China. The target sequence of si-SUMO1P3 was 5′-TGGCCCTGATGTTCTAGCATGTGAT-3′. The cells were cultured 24 h prior to transfection. Then, the cells were transiently transfected with either SUMO1P3 siRNA or negative control siRNA using Lipofectamine 2000 Transfection Reagent (Invitrogen, Carlsbad, CA, USA) according to the manufacturer's instructions. After 48 h, cells transfected with siRNA were harvested for qRT-PCR.

### RNA extraction and quantitative real-time PCR

The total RNA of the tissue samples or the transfected cells were extracted using the Trizol reagent (Invitrogen, Carlsbad, CA, USA) according to the manufacturer's protocol. The concentration and purity of the total RNA were detected with UV spectrophotometer analysis at 260 nm and the electrophoresis detection showed good quality of purified RNA. cDNA was converted from total RNA by using SuperScript III (Invitrogen) according to the instructions. The primer sequences were as follows: SUMO1P3 primers^8^ forward: 5′-ACTGGGAATGGAGGAAGA-3′, reverse: 5′-TGAGAAAGGATTGAGGGAAAAG-3′; GAPDH primers forward: 5′-CGCTCTCTGCTCCTCCTGTTC-3′, reverse: 5′-ATCCGTTGACTCCGACCTTCAC-3′. Quantitative real-time PCR was performed by using the ABI PRISM 7000 Fluorescent Quantitative PCR System (Applied Biosystems, Foster City, CA, USA) according to the manufacturer's instructions. The average value in each triplicate was used to calculate the relative amount of SUMO1P3 using 2−ΔΔCt methods. Experiments were repeated at least three times.

### Cell counting Kit-8 assay

Cell proliferation was determined using *Cell Counting Kit-8* (Beyotime Inst Biotech, China) according to instructions. Briefly, 5 × 10^3^ cells/well were seeded in a 96-well flat-bottomed plate for 24 h, then transfected with si-SUMO1P3 or si-NC and cultured in normal medium. At 0, 24, 48 and 72h after transfection, 10 μl of CCK-8 (5mg/ml) was added to each well and the cells were cultured for 1 hour [28]. The absorbance in each well was finally determined at a wavelength of 450 nm using a microplate reader (Bio-Rad, Hercules, CA, USA). Experiments were repeated at least three times.

### Ethynyl-2-deoxyuridine (EdU) incorporation assay

Cell proliferation was also determined by Ethynyl-2-deoxyuridine incorporation assay using an EdU Apollo DNA in vitro kit (RIBOBIO, Guangzhou, China) following the manufacturer's instructions. Briefly, 5 × 10^3^ cells/well was seeded in a 96-well plate for 24 h, then transfected with si-SUMO1P3 or si-NC. At 48h after transfection, cells were incubated with 100 μl of 50 μM EdU per well for 2 h at 37°C. Then, the cells were fixed for 30 min at room temperature using 100μl of fixing buffer (4% polyformaldehyde containing PBS). Subsequently, the cells were incubated with 50μl of 2 mg/ml glycine for 5 min followed by washing with 100μl of PBS. After permeabilization with 0.5% TritonX, the cells were reacted with 1X Apollo solution for 30 min at room temperature in the dark. After that, cells were incubated with 100μl of 1X Hoechst 33342 solution for 30 min at room temperature in the dark followed by washing with 100μl of PBS [29]. The cells were then visualized under a fluorescence microscopy. Experiments were repeated at least three times.

### Caspase-3 ELISA assay

Cell apoptosis was determined by ELISA assay. Cell Caspase-3 activity was measured using the Caspase-3 Colorimetric Assay kit (Abcam, Cambridge, UK) according to the manufacturer's protocol at 48 hours after transfection, respectively[29]. Experiments were repeated at least three times in duplicates.

### Hoechst 33342 staining assay

Apoptotic cells induced by SUMO1P3 silencing were also observed by using the Hoechst 33258 staining kit (Life, Eugene, OR, USA) according to the manufacturer's instructions. Briefly, 5 × 10^4^ cells/well was seeded in a 12-well plate for 24 h, then transfected with siRNA. At 48h after transfection, the cells were fixed for 30 min at room temperature using 100μl of fixing buffer (4% polyformaldehyde containing PBS). Subsequently, cells were incubated with 100μl of 1X Hoechst 33342 solution for 30 min at room temperature in the dark followed by washing with 100μl of PBS [30]. The cells were then visualized under a fluorescence microscopy. Experiments were repeated at least three times.

### Flow cytometry assay

Cell apoptosis was also determined by flow cytometry. After 48 h, cells transfected with siRNA were harvested for flow cytometry assay. After double staining with FITC-Annexin V and PI according to the manufacturer's instructions, cell apoptosis was determined by using flow cytometry (EPICS, XL-4, Beckman, CA, USA), respectively. Experiments were repeated at least three times in duplicates [31].

### Wound healing assay

Cell motility was determined by wound healing assay. At 24 h post transfection, a wound field was created using a sterile 200 μl pipette tip in about 90% confluent cells. The cells were incubated for 24 h at 37°C, and then the migration of cells was monitored with a digital camera system. The cell migration distance (μm) was calculated by the software program HMIAS-2000 [32]. Experiments were repeated at least three times.

### Transwell assay

The cell motility assay were also performed using a transwell insert (8 μm, Corning). 24 h after transfection, 5×104 cells were first starved in 200 ml serumfree medium and then placed in the uncoated dishes [33]. The lower chamber was filled with 500 ml of complete medium. The cells were incubated for 48 h at 37°C, and then the cells that had migrated to the bottom surface of the filter membrane were stained with 0.5% crystal violet solution and photographed in five preset fields per insert. The results represented the average of three independent experiments.

### Statistical analyses

All experimental data from three independent experiments were analyzed by Student's t-test or ANOVA and results were expressed as mean ± SD. P-values of less than 0.05 were considered to be statistically significant. All statistical tests were conducted by SPSS version 19.0 software (SPSS Inc. Chicago, IL, USA).
